# Serotonergic dysfunctions and abnormal iron metabolism: Relevant to mental fatigue of Parkinson disease

**DOI:** 10.1038/s41598-016-0018-z

**Published:** 2016-12-21

**Authors:** Li-Jun Zuo, Shu-Yang Yu, Yang Hu, Fang Wang, Ying-Shan Piao, Teng-Hong Lian, Qiu-Jin Yu, Rui-Dan Wang, Li-Xia Li, Peng Guo, Yang Du, Rong-Yan Zhu, Zhao Jin, Ya-Jie Wang, Xiao-Min Wang, Piu Chan, Sheng-Di Chen, Yong-Jun Wang, Wei Zhang

**Affiliations:** 1 Department of Neurology, Beijing Tiantan Hospital, Capital Medical University, Beijing, 100050 China; 2 Department of Geriatrics, Beijing Tiantan Hospital, Capital Medical University, Beijing, 100050 China; 3 Core Laboratory for Clinical Medical Research, Beijing Tiantan Hospital, Capital Medical University, Beijing, 100050 China; 4 Department of Neurobiology and Physiology, Capital Medical University, Beijing, 100069 China; 5 Key Laboratory for Neurodegenerative Disorders of the Ministry of Education, Capital Medical University, Beijing, 100069 China; 6 Beijing Key Laboratory on Parkinson Disease, Beijing, 100053 China; 7 Beijing Institute for Brain Disorders, Capital Medical University, Beijing, 100069 China; 8 Department of Neurobiology, Beijing Xuanwu Hospital, Capital Medical University, Beijing, 100053 China; 9 Department of Neurobiology, Ruijin Hospital Affiliated to Shanghai Jiaotong University School of Medicine, Shanghai, 200025 China; 10 China National Clinical Research Center for Neurological Diseases, Beijing, 100050 China

## Abstract

Fatigue is a very common non-motor symptom in Parkinson disease (PD) patients. It included physical fatigue and mental fatigue. The potential mechanisms of mental fatigue involving serotonergic dysfunction and abnormal iron metabolism are still unknown. Therefore, we evaluated the fatigue symptoms, classified PD patients into fatigue group and non-fatigue group, and detected the levels of serotonin, iron and related proteins in CSF and serum. In CSF, 5-HT level is significantly decreased and the levels of iron and transferrin are dramatically increased in fatigue group. In fatigue group, mental fatigue score is negatively correlated with 5-HT level in CSF, and positively correlated with the scores of depression and excessive daytime sleepiness, and disease duration, also, mental fatigue is positively correlated with the levels of iron and transferrin in CSF. Transferrin level is negatively correlated with 5-HT level in CSF. In serum, the levels of 5-HT and transferrin are markedly decreased in fatigue group; mental fatigue score exhibits a negative correlation with 5-HT level. Thus serotonin dysfunction in both central and peripheral systems may be correlated with mental fatigue through abnormal iron metabolism. Depression, excessive daytime sleepiness and disease duration were the risk factors for mental fatigue of PD.

## Introduction

Fatigue is one of the most common and disabling symptoms in Parkinson’s disease (PD) with high prevalence of 58.1%^1^. As Kluger proposes criteria for diagnosis of PD-related fatigue, patients must report significantly diminished energy levels or increased perceptions of effort that are disproportionate to attempted activities or general activity level. Symptoms must be present for most of the day every day or nearly every day during the previous month, adding other 4 or more additional symptoms^2^. Fatigue in PD contains mental fatigue and physical fatigue, which can be identified by Fatigue Scale-14 (FS-14). Mental fatigue occurs after sustained intellectual activity or emotional tension^3^. Physical fatigue is a sense of exhaustion caused by repeated muscular contraction or continuous physical activity^4^. Fatigue in PD mainly manifested mental fatigue^5^. So mental fatigue might present the main characteristic of fatigue in PD. Yet, there is no study exploring the underlying mechanism about mental fatigue in PD. Several studies reported that fatigue was related to depression and sleep disorders, however, there is no further study investigating the correlation of mental fatigue with depression and sleep disorders.

[N,N-dimethyl-2-(2-amino-4-cyanophenylthio) benzylamine] (^11^C-DASB) PET reveals that fatigue in PD patients is related to striatal and limbic serotonergic dysfunction^6^. There is no studies directly investigating the relationship between 5-hydroxytryptamine (5-HT) level both in CSF and serum and mental fatigue score in PD patients.

Several autopsy reports showed iron deposition in substantia nigra (SN) in PD patients. Studies reported that PD patients had hyperechogenicity in SN by transcranial sonography (TCS)^7^ and iron deposition was mainly in SN pars compacta (SNpc) by susceptibility weighted imaging (SWI)^8^. Iron-related neurodegeneration could be attributed for the defects in its metabolism and/or homeostasis and subsequent accumulation in the specific brain regions. For example, the levels of transferrin, an iron metabolism-related protein, in brains of PD subjects were remarkably increased comparing with normal control subjects^9^. Studies implied that gene mutations in the iron metabolism-related proteins, such as transferrin^10^ and ferritin^11^ were related to the development of PD, indicating that abnormal iron and related proteins in brain participate in the pathogenesis of PD. However, there is no study detecting the relationship between iron metabolism in CSF and serum and mental fatigue in PD patients. Moreover, the relationship among 5-HT, iron metabolism and mental fatigue in PD is unknown.

In this study, in PD patients, we assessed mental fatigue by FS-14, detected the levels of 5-HT, iron and related proteins, including transferrin, lactoferrin and ferritin in CSF and serum, and analyzed the correlations among mental fatigue score and the levels of above factors, and attempted to figure out the mechanisms underlying mental fatigue relating 5-HT and iron metabolism in PD patients with fatigue.

## Methods

### Subjects

Patients with PD. PD patients were recruited from the neurodegenerative outpatient clinics in the Department of Geriatrics and Neurology, Beijing Tiantan Hospital, Capital Medical University. Demographic information including age, sex, disease severity and disease duration as well as levodopa equivalent daily doses was recorded. PD patients were diagnosed satisfying to the criteria of Parkinson’s UK Brain Bank^12^. PD patients with blood donation histories, systemic diseases including pulmonary disorders, anemia, heart failure, chronic liver/renal failure, hepatosis, severe hypothyroidism and diabetes were excluded. Female patients who had not been through menopause were not included in this study. PD patients with an Apathy Scale score of ≥14 were excluded^13^. This study consecutively recruited 530 PD patients. Of 530 PD patients, 4 patients with pulmonary disorders, 5 patients with severe hypothyroidism and 3 patients with heart failure were also excluded. Finally, 518 PD patients were recruited in this study.

Control subjects. Total 29 age-matched controls from Beijing Tiantan Hospital were selected based on the following criteria: (1) no essential tremor, PD, secondary parkinsonism, or Parkinson-plus syndrome; (2) no systemic diseases affecting fatigue or sleep, such as pulmonary disorders, heart failure, hypertension, diabetes, anemia, hepatosis, chronic liver/renal failure, severe hypothyroidism or epilepsy history; (3) no histories of blood donation; (4) no intracranial diseases; (5) no neurological symptoms and signs; (6) no obvious depression, apathy, cognitive impairment, or psychiatric symptoms; (7) no dysarthria or mental illness that affects expression; (8) no alcohol or drug abuse. Female controls were excluded who had not been through menopause in this study. The controls were also patients, but their diseases, such as peripheral neuropathy and headache caused by high intracranial pressure, were not related to and did not influence the results of this investigation.

### Assessment of PD

#### Assessment of fatigue

The Fatigue Severity Scale (FSS) is a “recommended” fatigue scale (both for screening and severity rating) and has good psychometric features (discrimination fatigued patients from non-fatigued patients) in PD^14^. It is a self-rating fatigue scale with 9 items, and encompasses several aspects of fatigue. Patients were asked to evaluate how each item described their fatigue level from 1 (strongly disagree) to 7 (strongly agree). Total FSS score was obtained by dividing the sum of all item scores by 9. Patients with total FSS score >4 points and ≤4 points were classified into the fatigue group and non-fatigue group, respectively^14^.

FS-14 is a 14-item self-rating scale for fatigue evaluation. Items 1–8 and 9–14 of FS-14 reflect physical fatigue and mental fatigue, respectively. Higher total score of FS-14 indicates severer fatigue^15^. The sensitivity and specificity of FS-14 are 75.5 % and 74.5%, respectively.

This study has been approved by Beijing Tiantan Hospital review board (KY2013-003-03). Written informed consent was obtained from all participating subjects. This study was performed according to the guidelines of Capital Medical University, which abides by the Helsinki Declaration on ethical principles for medical research involving human subjects.

### Clinical assessments of motor symptoms and non-motor symptoms

The severity of PD was evaluated by the Hoehn and Yahr (H-Y) stage. Motor symptoms were evaluated by Unified Parkinson’s Disease Rating Scale (UPDRS) III, in which items 20 and 21 were for tremor, item 22 was for rigidity, items 23–26 and 31 were for bradykinesia, and items 27–30 were for postural and gait abnormalities. The score for each motor symptom was calculated by summing up the score for the relevant items in UPDRS III. Non-motor symptoms were evaluated by using the following scales: Hamilton Anxiety Scale (HAMA) (14 items) for anxiety, Hamilton Depression Scale (HAMD) (24 items) for depression, Mini-Mental State Examination (MMSE) for cognitive impairment, Pittsburgh Sleep Quality Index (PSQI) for total sleep disorders, and Epworth Sleepiness Scale (ESS) for excessive daytime sleepiness.

### CSF and serum sample collection

Patients were requested to withhold anti-parkinsonian drugs for 12–14 hours if their condition allowed. Total 2 ml venous whole blood was collected and 3 ml CSF was taken in a polypropylene tube between 7 a.m. and 10 a.m. under fasting condition through lumbar puncture. Approximately 0.5 ml volume of CSF and serum were aliquotted into separate Nunc cryotubes and kept frozen at −80 °C until ready for assay. Each aliquot dedicated for each measure to avoid freeze-thawing and potential degradation of protein.

### Detection of the level of 5-HT in CSF and serum

The level of 5-HT in CSF and serum from PD patients were measured by high performance liquid chromatography (HPLC). Henomenex 150 * 2 mm, 150 * 3 mm chromatographic columns and LC-MS-MS 6410 chromatographic instrument were from Agilent Company (USA), and standard sample was from Sigma Company (USA).

### Detection of the levels of iron and related proteins in CSF and serum

The levels of iron and related proteins, including iron, transferrin, ferritin and lactoferrin in CSF and serum from PD patients were detected by Enzyme Linked Immunosorbent Assay (ELISA). CSB-E08831h kit for lactoferrin was from Wuhan Huamei Biological Limited Company (Wuhan, China). Ab83366 kit for iron, Ab108837 kit for ferritin and Ab108911 kit for transferrin were from Abcam Company (Cambridge, United Kindom).

### Data analyses

Statistical analyses were performed with SPSS Statistics 20.0 (IBM Corporation, New York, USA).

Demographics information, motor symptoms and non-motor symptoms were compared between fatigue and non-fatigue groups. The levels of 5-HT, iron and related proteins in CSF and serum were compared among control, non-fatigue and fatigue groups.

Continuous variables, if they were normally distributed, were presented as means ± standard deviations and compared by ANOVA test. Bonferroni correction was performed in further comparisons between two groups. P value was significant when it was <0.017. Continuous variables, if they were not normally distributed, were presented as median (quartile) and compared by nonparametric test. P value was significant when it was <0.017 in further comparisons between two groups. Discrete variables were compared by Chi square test.

Spearman correlation analyses were made between mental fatigue score and 5-HT level in CSF and serum, between mental fatigue score and the levels of iron and related proteins in CSF and serum, among the levels of iron and tranferrin in CSF and age, disease duration, the scores of UPDRS III, tremor, rigidity, bradykinesia, postural and gait abnormalities, HAMD, HAMA and ESS, and between 5-HT level and the levels of iron and related proteins in CSF in PD with fatigue group.

FS-14 lacked validation and had conflation of fatigue with sleepiness. Therefore, we added ESS for evaluation of excessive daytime sleepiness. Fatigue group scored significantly higher on the scores of UPDRS III, tremor, bradykinesia, rigidity, postural and gait abnormalities, HAMA, HAMD and ESS and had significantly advanced H-Y stage than non-fatigue group. Previous study reported that fatigue in PD patients was related to longer disease duration^16^. Therefore, in the multiple linear regression models, age, sex, disease duration, H-Y stage, the scores of UPDRS III, tremor, bradykinesia, rigidity, postural and gait abnormalities, HAMD, HAMA, ESS and 5-HT level in CSF were set as independent variables, and mental fatigue score was set as a dependent variable. There was no significant difference in PSQI between fatigue and non-fatigue groups. Therefore, we did not include PSQI in the multiple linear regression model.

## Results

### Frequency and assessment of fatigue in PD patients

Among the 518 PD patients, 250 cases (52.12%) were male and 268 (47.88%) were female. The average score of mental fatigue in fatigue and non-fatigue groups was 7.00 (6.00~8.00) and 4.00 (2.00~6.00) points, respectively. The disease duration varied from 3 month to 33 years, with a median of 2.5 years [interquartile range (IQR): 4.0 years]. The demographic characteristics were listed in Table 1, Supplemental Table 1 and Supplemental Table 2.

**Table 1:** Demographics information, motor and non-motor symptoms in non-fatigue and fatigue groups.

	Non-fatigue group (213 cases)	Fatigue group (305 cases)	P value
Age (years, mean ± SD)	60.85 ± 10.44	61.73 ± 10.07	0.89
Male/Total [cases/total (%)]	108/213 (50.70%)	162/305 (53.11%)	0.79
Disease duration [years, median (quartile)]	2.00 (1.00~4.00)	3.00 (1.00~6.00)	0.23
Hoehn-Yahr stage (stage, mean ± SD)	1.80 ± 0.72	2.19 ± 0.83	0.01*
Levodopa equivalent dose (mg, mean ± SD)	319.13 ± 107.98	322.79 ± 113.54	0.72
UPDRS III [scores, median (quartile)]	18.00 (11.50~25.50)	27.50 (19.00~36.00)	0.00**
Tremor	3.00 (2.00~6.00)	5.00 (2.00~8.00)	0.02*
Rigidity	3.00 (1.00~6.00)	5.00 (2.00~8.00)	0.00**
Bradykinesia	2.50 (1.75~4.00)	4.00 (2.00~6.00)	0.00**
Postural and gait abnormalities	7.00 (4.00~12.00)	11.00 (6.00~16.00)	0.00**
Mental fatigue [scores, median (quartile)]	4.00 (2.00~6.00)	7.00 (6.00~8.00)	0.00**
Total fatigue [scores, median (quartile)]	6.00 (4.00~9.00)	11.00 (9.00~13.00)	0.00**
HAMA [scores, median (quartile)]	5.00 (2.00~9.00)	11.00 (6.00~18.00)	0.00**
HAMD [scores, median (quartile)]	5.00 (3.00~11.00)	15.00 (8.00~20.00)	0.00**
MMSE [scores, mean ± SD]	26.93 ± 3.61	26.37 ± 3.33	0.78
PSQI [scores, mean ± SD]	7.12 ± 3.00	8.64 ± 4.74	0.21
ESS [scores, mean ± SD]	4.09 ± 2.96	5.54 ± 4.31	0.00*

HAMD = Hamilton Depression Scale (24 items); HAMA = Hamilton Anxiety Scale (14 items); UPDRS = Unified Parkinson’s Disease Rating Scale; MMSE = mini-mental state examination; PSQI = Pittsburgh Sleep Quality Index. *P < 0.05, **P < 0.01.

In the 518 PD patients, 80 cases (15.44%) had fatigue before the onset of motor symptoms. Fatigue group showed more advanced H-Y stage, higher total UPDRS III scores and higher scores of tremor, bradykinesia, rigidity, postural and gait abnormalities according to UPDRS III when compared with non-fatigue group. Fatigue group also scored drastically higher on HAMD, HAMA and ESS than non-fatigue group, suggesting that individuals in the fatigue group have severer depression, anxiety and excessive daytime sleepiness than those in non-fatigue group. Fatigue group and non-fatigue group did not differ in terms of demographic information, such as age, sex, disease duration and levodopa equivalent daily dose (Table 1, Supplemental Table 1 and Supplemental Table 2).

### Relationships among mental fatigue score, the levels of 5-HT, iron and related proteins in CSF

#### Relationship between mental fatigue score and 5-HT level in CSF

The 5-HT level in CSF was compared among control, fatigue and non-fatigue groups (Table 2). The 5-HT level in CSF in fatigue group was prominently lower than that in control and non-fatigue groups. Further analysis indicated that mental fatigue score increased with the decreased 5-HT level (r = −0.37, P < 0.05) in CSF in PD with fatigue group.

**Table 2:** The levels of 5-HT, iron and metabolism-related proteins in CSF among control, non-fatigue and fatigue groups.

	Control group (29 cases)	Non-fatigue group(59 cases)	Fatigue group(63 cases)	P1	P2	P3
**Neurotransmitters**						
5-HT [ng/mL, median (quartile)]	10.617 (5.732~115.828)	8.934 (4.421~107.512)	5.546 (4.312~338.01)	0.094	0.000**	0.000**
**Iron and metabolism-related proteins**						
Iron [nmol/mL, median (quartile)]	0.380 (0.263~0.612)	0.411 (0.261~0.8254)	0.632 (0.321~0.845)	0.354	0.000**	0.004**
Transferrin [ug/ml, median (quartile)]	0.079 (0.062~0.083)	0.104 (0.084~0.123)	0.192 (0.073~0.214)	0.001**	0.000**	0.003**
Lactoferrin (ug/ml, mean ± SD)	148.471 ± 65.153	138.822 ± 61.371	134.295 ± 53.764	0.231	0.114	0.614
Ferritin [ng/ml, median (quartile)]	5.291 (2.592~20.723)	5.771 (3.043~14.221)	5.854 (3.632~17.231)	0.783	0.63	0.853

5-HT = serotonin; P1: non-fatigue group vs. control group; P2: fatigue group vs. control group, P3: non-fatigue group vs. fatigue group. **P < 0.01.

#### Relationship between mental fatigue score and the levels of iron and related proteins in CSF

The levels of iron, transferrin, ferritin and lactoferrin in CSF were compared among control, fatigue and non-fatigue groups (Table 2). The levels of iron and transferrin in CSF in fatigue group were prominently higher than those in control and non-fatigue groups. The transferrin level in CSF in non-fatigue was strikingly higher than that in control group. Correlation analyses demonstrated that mental fatigue score increased with the elevated levels of iron (r = 0.381, P < 0.05) and transferrin (r = 0.435, P < 0.05) in CSF in PD with fatigue group.

### Relationship among the levels of 5-HT, iron and transferrin in CSF, age, age of onset, disease duration, the scores of UPDRS III, tremor, rigidity, bradykinesia, postural and gait abnormalities, depression, anxiety and excessive daytime sleepiness in PD with fatigue group

Analyses of the correlations of the 5-HT level in CSF in PD with fatigue group with age, age of onset, disease duration, the scores of UPDRS III, tremor, rigidity, bradykinesia and postural and gait abnormalities, depression, anxiety and excessive daytime sleepiness implied that 5-HT level was negatively correlated with the scores of rigidity (r = −0.31, P < 0.05) and HAMD (r = −0.72, P < 0.05).

Analyses of the correlations of iron level in CSF in PD with fatigue group with age, age of onset, disease duration, the scores of UPDRS III, tremor, rigidity, bradykinesia and postural and gait abnormalities, depression, anxiety and excessive daytime sleepiness revealed that iron level in CSF was positively correlated with the scores of rigidity (r = 0.76, P < 0.05) and bradykinesia (r = 0.21, P < 0.05).

Analyses of the correlations of transferrin level in CSF with age, age of onset, disease duration, the scores of UPDRS III, tremor, rigidity, bradykinesia and postural and gait abnormalities, depression, anxiety and excessive daytime sleepiness indicated no significant correlations.

### Relationship between 5-HT level and the levels of iron and related proteins in CSF in PD with fatigue group

Further analyses indicated that 5-HT level decreased with the increased transferrin level (r = −0.429, P < 0.05) in CSF in PD with fatigue group (Table 3). In the multiple linear regression models, we still found that 5-HT level in CSF was significantly and negatively correlated with transferrin level (r = −0.733, P < 0.05) after adjusting for confounders in PD with fatigue group.

**Table 3:** Correlation between the levels of 5-HT and transferrin in CSF in PD with fatigue group.

Neurotransmitters (ng/mL)	Iron and related proteins (ug/ml)	R	P value
5-HT	transferrin	**−0.429**	0.006**

5-HT = serotonin; **P < 0.01.

### Influencing factors of mental fatigue in PD group

Multiple linear regression model (Type III) was established, in which mental fatigue in PD with fatigue group was set as dependent variable, whereas age, sex, disease duration, H-Y stage, the scores of UPDRS III, tremor, bradykinesia, rigidity, postural and gait abnormalities, HAMD, HAMA, ESS and 5-HT level in CSF were et as independent variables. The results indicated that 5-HT level in CSF, the scores of HAMD and ESS, and disease duration were the influencing factors for mental fatigue score in PD group (regression coefficient =−0.557, 0.627, 0.676 and 0.447, P < 0.05), whereas the scores of HAMA, UPDRS III, tremor, bradykinesia, rigidity, postural and gait abnormalities, age, sex and H-Y stage did not enter the regression equation (Supplemental Table 3).

### Relationship between mental fatigue score and the levels of 5-HT, iron and related proteins in serum

#### Relationship between mental fatigue score and 5-HT level in serum

5-HT level in serum was compared among control, fatigue and non-fatigue groups (Table 4). The decreased 5-HT level in serum was observed in fatigue and non-fatigue groups comparing with control group. Further analysis showed mental fatigue score increased with the reduced 5-HT level (r = −0.344, P = 0.012) in serum in PD with fatigue group.

**Table 4:** The levels of 5-HT, iron and metabolism-related proteins in serum among control, non-fatigue and fatigue groups.

	Control group(29 cases)	Non-fatigue group(125 cases)	Fatigue group (145 cases)	P1	P2	P3
**Neurotransmitters**						
5-HT [ng/mL, median (quartile)]	415.812 (319.142~522.327)	217.323 (126.017~289.543)	230.619 (135.815~305.719)	0.001**	0.000**	0.635
**Iron and metabolism-related proteins**						
Iron [nmol/ml, mean ± SD]	3.322 (2.624~4.861)	3.011 (2.113~4.324)	3.121 (2.871~4.434)	0.259	0.382	0.624
Transferrin [ug/ml, median (quartile)]	0.145 (0.177~0.564)	0.089 (0.069~0.094)	0.076 (0.066~0.084)	0.000**	0.000**	0.023
Lactoferrin [ug/ml, median (quartile)]	51.591 (45.214~86.022)	51.711 (47.282~88.894)	50.882 (44.981~87.805)	0.768	0.456	0.647
Ferritin [ng/ml, median (quartile)]	16.775 (6.241~48.983)	15.772 (7.043~41.223)	16.401 (7.455~43.742)	0.701	0.816	0.984

5-HT = serotonin; P1: non-fatigue group vs. control group; P2: fatigue group vs. control group, P3: non-fatigue group vs. fatigue group. **P < 0.01.

#### Relationship between mental fatigue score and the levels of iron and related proteins in serum in PD with fatigue group

The levels of iron, transferrin, ferritin and lactoferrin in serum were compared among control, fatigue and non-fatigue groups (Table 4). The data revealed that transferrin level in serum in fatigue group was prominently decreased comparing with non-fatigue and control groups. Further analyses implied no relationship between mental fatigue score and the levels of iron and related proteins in serum.

#### Relationship between 5-HT level and the levels of iron and related proteins in serum in PD with fatigue group

Correlation analyses were made between 5-HT level and the levels of iron and related proteins in serum. The data did not indicate any correlation among them (r = 0.51, P > 0.05).

## Discussion

In this study, 58.88% of total PD patients had fatigue, indicating that fatigue is a very common non-motor symptom in PD patients. The frequency of PD with fatigue in this investigation was higher than that in previous report, which might be accounted for the differences in H-Y stage and disease duration of the patients, and scales used for evaluating fatigue^1^. Eighty out of 518 PD patients (15.44%) were with fatigue prior to the appearance of motor symptoms, supporting that fatigue may be one of prodromal symptoms of PD. One study showed that fatigue frequently occurred in the 2 to 10 years premotor period^17^. Patients with fatigue might have a high risk of 1.56 to develop PD^18^. Fatigue could help to identify individuals at the earliest stages of PD. PD patients with fatigue in the present study showed a more advanced H-Y stage, severer motor symptoms and non-motor symptoms indicated by higher scores of total UPDRS III, HAMA, HAMD and ESS (Table 1, Supplemental Table 1 and Supplemental Table 2). Importantly, further analyses of each motor symptom in the PD patients revealed that the score of each motor symptom, including tremor, bradykinesia, rigidity and postural and gait abnormalities, in the fatigue group was significantly higher than that in the non-fatigue group (Table 1, Supplemental Table 1 and Supplemental Table 2), illustrating that fatigue worsens with disease progression^16^. Previous studies have reported that bradykinesia, rigidity^19^ and postural and gait abnormalities^20,21^ were related to fatigue of PD. The present study revealed that tremor was related to fatigue of PD. It might be that both tremor and fatigue have the same central origin.

In the normal brain, the serotonergic system originates from the raphe nuclei and projects on the basal ganglia and other cortical and subcortical structures^22^. Tremor is linked to the parkisonian pathology in the raphe nuclei, interrupting serotonergic output to cerebellar, thalamic and basal ganglia structures^23,24^. Study showed that fatigue patients in PD had lower serotonin transporter availability in striatal structures compared to non-fatigued patients^6^. It implied that both tremor and fatigue were related to serotonergic dysfunction. This study also found that fatigue became severe with disease duration, which was consistent with previous study, suggesting that PD patients with fatigue had a longer disease duration than those without fatigue^16^.

There were several study investigating the relationship between depression and fatigue in PD^2,25–27^. Multiple, but not all, studies in PD showed a significant correlation between depression and fatigue^16,25,28^. It might be speculated that these two symptoms shared the same pathophysiologic mechanisms. Depression was reported to be associated with decreased levels of 5-HT and its metabolite-5-hydroxyindoleacetic acid (5-HIAA) in CSF in PD patients^29^. In this study, we for the first time found that depression was significantly and positively correlated with mental fatigue in multiple linear regression in PD with fatigue group (r = 0.627, P < 0.05). We also observed that depression was negatively correlated with 5-HT level in CSF (r = −0.72, P < 0.05) and mental fatigue was negatively correlated with 5-HT level in CSF (r = −0.37, P < 0.05) in PD with fatigue group. Thus, we speculated that depression was related to mental fatigue in symptomatology, with a common neurochemical basis related to 5-HT.

Several previous studies have reported that fatigue was related to excessive daytime sleepiness^16,30^. Preclinical studies suggested the brainstem raphe nuclei contributed to an ascending arousal system that promoted wakefulness and prevented excessive daytime sleepiness^31^. Therefore, excessive daytime sleepiness and fatigue in PD were commonly related to the dysfunction of raphe nuclei in brainstem. Both of the two non-motor symptoms started PD pathology in the raphe nuclei^32^. In addition, a previous study reported that sleep-wake disturbances were related to fatigue and excessive daytime sleepiness. The neurotransmitter serotonin governs sleep-wake behavior^33^, and a reduction of serotonin transporters was reported to be correlated with fatigue. In this study, we found that fatigue group had severer excessive daytime sleepiness indicated by higher ESS score than non-fatigue group. Furtherly, we for the first time showed that mental fatigue score was related to excessive daytime sleepiness (r = 0.676, P < 0.05). These findings suggested that mental fatigue and excessive daytime sleepiness might share common underlying pathophysiology and were both related to serotoninergic functions.

PSQI is a comprehensive scale evaluating many aspects of sleep disorders. In this study, PSQI score in fatigue and non-fatigue groups was not different, and there was no correlation between the scores of mental fatigue and total sleep disorders. These data demonstrated that mental fatigue was highly correlated with excessive daytime sleepiness, but not other aspect of sleep disorders in PD patients.

To our knowledge, this is the largest study assessing 5-HT level in CSF in PD patients, and exploring the relationship between 5-HT level in CSF and mental fatigue score. Currently, growing evidence suggests that PD does not solely affect the dopaminergic system, but also serotonergic system with the data from biochemical, animal, postmortem, and functional imaging studies^34^. Even in early stage of PD patients, it was also observed reduced serotonin transporter availability^35^. In this study, we observed that 5-HT level in CSF in fatigue group was prominently lower than that in control and non-fatigue groups (Table 2). Profoundly, we found that mental fatigue score was associated with declined 5-HT level in CSF (Supplemental Table 3), implying a potential dysfunction of serotonergic system in PD patients with mental fatigue. In brain, the serotonergic system originates from the brainstem. When Lewy bodies appear in Braak stage 2 in the lower raphe nuclei and locus coeruleus, PD patients manifest with fatigue^36^. CSF is an optimal and objective source for allowing us to test and monitor the change of 5-HT level in the serotonergic system-containing brain regions in PD patients. We for the first time demonstrated that decreased 5-HT in CSF was highly correlated with mental fatigue in PD, implying that decreased 5-HT level in CSF might be a predictor for mental fatigue of PD. Recent study showed that an additional administration of 5-HT might relieve fatigue in PD patients^26^, implying that declined 5-HT level in CSF could affected mental fatigue.

There were rare studies investigating 5-HT level in serum in PD patients. 5-HT level in serum is balanced by 5-HT secretion, catabolism and platelet uptake mechanisms^37^. In this study, we found the decreased level of 5-HT in serum in both fatigue and non-fatigue groups comparing with the control group (Table 4). Further analysis showed that mental fatigue score increased with the decline of 5-HT level in serum in PD group. The decreased 5-HT level in serum was consistent with its level in CSF in PD patients, suggesting that serum could be a reasonable alternative to CSF when measuring 5-HT level since it was much less invasive.

We furtherly explored the mechanism for the decline of 5-HT level in CSF in PD patients with mental fatigue. A recent study showed that 5-(Nmethyl-N-propargyaminomethyl)-8-hydroxyquinoline (M30), an iron chelator, could increase 5-HT level in the brain of PD rat^38^. By far, no study focused on the relationship between iron and related proteins and mental fatigue of PD. In this study, the levels of transferrin in CSF in fatigue group and non-fatigue group were all prominently higher than that in control group (Table 2), implying dysfunction of iron transportation in PD brain. At the same time, the levels of iron and transferrin in CSF in fatigue group were prominently higher than those in non-fatigue group (Table 2), and mental fatigue score increased with the elevated levels of iron and transferrin in CSF in PD group, illustrating that compromised iron transportation and subsequent excessive iron deposition in brain might be the potential mechanism of PD with mental fatigue. Our study firstly found that increased iron level in CSF was related to mental fatigue. In the brain, iron in brain interstitium might bind to large molecules, such as transferrin, and then was transported into neurons. In the periphery, excessive intake of exogenous iron might induce redundant iron deposition in the brain through blood-brain barrier (BBB)^39^. Excessive iron deposits in brain region, such as raphe nuclei, is related to mental fatigue, resulting in symptom of fatigue. These results indicated a potential role of abnormal iron metabolism on mental fatigue in PD patients, and iron and transferrin might be the potential indicators for mental fatigue in PD patients.

In this study, transferrin levels in serum in the fatigue group and non-fatigue group were reduced comparing with that in the control group (Table 4). More transferrin in serum could transfer into brain through compromised BBB, and thus it might participate in the pathogenesis of PD^40^. Transferrin was the main receptor-mediated transporter of iron from periphery to brain across BBB and a transporter of iron throughout the brain^41^. Our previous work has found a decreased transferrin level in serum in PD patients with sleep disorders^42^, as well as PD patients with rapid eye movement-sleep behavior disorder (RBD)^43^. Hence, we speculated that BBB of PD patients might be more seriously damaged than that of control group, allowing the entry of transferrin from periphery to brain enormously, resulting in abnormal iron storage, transportation and accumulation in the brain region related to mental fatigue. However, further analyses showed no relationship between mental fatigue score and the levels of iron and related proteins in serum since their levels in the peripheral system might be affected by some uncontrolled elements, such as exogenous intake and viscera storage etc.

All PD patients recruited in this study came from the Department of Geriatrics and Neurology in Beijing Tiantan Hospital, the China National Clinical Research Center for Neurological Diseases. In Department of Geriatrics and Neurology, more than 80% patients were from the whole country. Although the patients in this study came from one center, 518 PD patients were from 28 out of 32 provinces and municipalities of China, roughly representing PD patients in China.

In summary, the frequency of fatigue in PD patients was 58.88%. Fatigue group had more advanced disease stage, severer motor symptoms, including tremor, bradykinesia, rigidity, postural and gait abnormalities, and severer non-motor symptoms, such as depression, anxiety, excessive daytime sleepiness, etc. Depression, anxiety, excessive daytime sleepiness and disease duration were the risk factors for mental fatigue in PD. Decreased 5-HT in CSF was closely correlated with mental fatigue in PD patients. The elevated levels of iron and transferrin in CSF were significantly correlated with mental fatigue in PD patients, which might result from the translocation of transferrin from peripheral system to brain. Overloaded iron might contribute to 5-HT dysfunction in brain region related to mental fatigue in PD patients. Thus, 5-HT reuptake inhibitors and iron chelator might serve as novel targets of drug development for mental fatigue of PD.

This study has some limitations. Firstly, it was a cross-sectional study, therefore, causal relationships between the levels of 5-HT and transferrin in the CSF of PD patients with mental fatigue was not be determined. Secondly, FS-14 scale has limitation in specificity for PD because of lack of validation and conflation of fatigue and sleepiness. 16-item self-report Parkinson Fatigue Scale (FS-16)^44^ will be needed in the future study to confirm the results of the present study. Thirdly, this study did not include PD patients with both fatigue and moderate or severe depression. Lastly, the data of PD patients are only from one center, it needs a large, nationwide and multicentric study to further investigate the mechanism of PD fatigue in the future.

## Electronic supplementary material

Supplementary Dataset 1
