# Supplementary material for: Serotonergic dysfunctions and abnormal iron metabolism: Relevant to mental fatigue of Parkinson disease
Source: Sci Rep. 2016 Dec 21;6:19. doi: 10.1038/s41598-016-0018-z (PMC5431345; doi:10.1038/s41598-016-0018-z)
Supplement: Supplementary file 1 — Supplementary Dataset 1 [file 41598_2016_18_MOESM1_ESM.pdf]

Serotonergic dysfunctions and abnormal iron metabolism:
Relevant to mental fatigue of Parkinson disease

Li-Jun ZUO MD[1], Shu-Yang YU MD[2], Yang HU MD[2], Fang WANG MD[1], Ying-Shan PIAO MD, PhD[2], Teng-Hong LIAN MD[1], Qiu-Jin YU MD[1], Rui-Dan WANG MD[1], Li-Xia LI MD[2], Peng GUO MD[2], Yang DU MD[1], Rong-Yan ZHU MD[1], Zhao JIN MD[1], Ya-Jie WANG PhD[6], Xiao-Min WANG PhD[7], Piu CHAN MD, PhD[4,8], Sheng-Di CHEN MD, PhD[9], Yong-Jun WANG MD[1,3], Wei ZHANG MD, PhD[2,1,3,4,5]

Supplemental Table 1 Demographics information, motor symptoms and non-motor symptoms in non-fatigue and fatigue groups with CSF collected

	Non-fatigue group (59 cases)	Fatigue group (63 cases)	P value
Age	60.37±10.45	61.29±9.93	0.92
Male/Total [cases/total (%)]	30/59（50.85%）	34/63（53.97%）	0.86
Disease duration [years, median (quartile)]	2.00（1.00~3.00）	3.00（1.00~5.50）	0.33
Hoehn-Yahr stage [stage, mean ±SD]	1.75±0.89	2.12±0.73	**0.03***
Levodopa equivalent dose(mg, mean ±SD)	310.23±109.45	326.87±112.36	0.54
UPDRS III [points, median (quartile)]	19.00（12.50~26.00）	28.50（19.00~36.00）	**0.00****
Tremor	4.00（2.00~7.00）	4.00（2.00~6.00）	**0.02***
Rigidity	3.00（2.00~5.50）	5.00（2.00~9.00）	**0.00****
Bradykinesia	2.00（1.00~4.00）	4.00（2.00~6.00）	**0.00****
Postural and gait abnormalities	7.00（4.00~13.00）	10.00（6.00~15.00）	**0.00****
Mental fatigue [scores, median (quartile)]	4.00（2.00~5.50）	7.00 （6.00~8.50）	**0.00****
Total fatigue [scores, median (quartile)]	6.00（3.00~8.50）	11.00（9.00~12.00）	**0.00****
HAMA [scores, median (quartile)]	5.00（3.00~9.00）	11.00（6.00~17.00）	**0.00****
HAMD [scores, median (quartile)]	5.00（4.00~11.00）	15.00（8.00~19.00）	**0.00****
ESS [scores, mean ± SD]	4.02±3.04	5.58±4.30	**0.00***
PSQI [scores, mean ± SD]	7.26 ± 3.21	8.15 ± 3.98	0.35
MMSE [scores, mean ± SD]	26.57±3.58	26.19 ± 3.28	0.82

HAMD=Hamilton Depression Scale (24 items); HAMA=Hamilton Anxiety Scale (14 items); UPDRS =Unified Parkinson's Disease Rating Scale; MMSE=Mini-mental State Examination; PSQI= Pittsburgh Sleep Quality Index.
*: P<0.05, ** P<0.01.

Supplemental Table 2 Demographics information, motor symptoms and non-motor symptoms in non-fatigue and fatigue groups with serum collected

	Non-fatigue group (125 cases)	Fatigue group (145 cases)	P value
Age	59.82±10.54	61.51±10.13	0.11
Male/Total [cases/total (%)]	30/59（50.85%）	34/63（53.97%）	0.86
Disease duration [years, median (quartile)]	2.00（1.00~5.00）	2.00（1.00~4.00）	0.38
H-Y stage [stage, mean ±SD]	1.79±0.84	2.12±0.65	**0.00****
Levodopa equivalent dose (mg, mean ±SD)	316.43±106.67	329.54±114.21	0.67
UPDRS III [points, median (quartile)]	18.25（12.00~26.00）	28.00（19.00~37.00）	**0.00****
Tremor	4.00（2.00~6.00）	3.00（2.00~5.00）	**0.045***
Rigidity	5.00（2.00~8.00）	3.00（0.00~3.00）	**0.00****
Bradykinesia	4.00（2.00~6.00）	2.00（1.00~4.00）	**0.00****
Postural and gait abnormalities	10.00（6.00~15.00）	7.00（4.00~12.00）	**0.00****
Mental fatigue [scores, median (quartile)]	2.00（1.00~4.00）	4.00（2.00~5.00）	**0.00****
Total fatigue [scores, median (quartile)]	6.00（3.00~9.00）	11.00（8.00~12.00）	**0.00****
HAMA [scores, median (quartile)]	5.00（2.00~8.00）	10.00（6.00~17.00）	**0.00****
HAMD [scores, median (quartile)]	5.00（3.00~12.00）	15.00（7.00~19.00）	**0.00****
ESS [scores, mean ± SD]	4.13±3.08	5.52±4.29	**0.00****
PSQI [scores, mean ± SD]	6.98 ± 3.19	8.21 ± 3.41	0.27
MMSE [scores, mean ± SD]	26.46 ± 4.09	26.28 ± 3.13	0.86

HAMD=Hamilton Depression Scale (24 items); HAMA=Hamilton Anxiety Scale (14 items); UPDRS =Unified Parkinson's Disease Rating Scale; MMSE=Mini-mental State Examination; PSQI= Pittsburgh Sleep Quality Index. *: P<0.05, ** P<0.01.

Supplemental Table 3 Influencing factors for mental fatigue in PD with fatigue group

	B	P value
Constant	4.68	0.001
Age	0.411	0.072
Gender	0.073	0.758
Disease duration	0.447	0.048*
Hoehn-Yahr stage	0.168	0.478
UPDRS III score	0.318	0.171
Tremor	-0.397	0.083
Rigidity	0.077	0.747
Bradykinesia	0.01	0.968
Postural and gait abnormalities	-0.016	0.947
HAMD score	0.627	0.003*
HAMA score	0.613	0.078
ESS score	0.676	0.001**
5-HT in CSF	-0.557	0.001**

Methods: Type Ⅲ regression; 5-HT=serotonin; HAMD=Hamilton Depression Scale (24 items); HAMA=Hamilton Anxiety Scale (14 items); UPDRS =Unified Parkinson's Disease Rating Scale; *$P < 0.05$.
